# Targeted long-read sequencing identified a causal structural variant in X-linked nephrogenic diabetes insipidus

**DOI:** 10.1186/s12920-024-01801-1

**Published:** 2024-01-22

**Authors:** Lukáš Strych, Monika Černá, Markéta Hejnalová, Tomáš Zavoral, Pavla Komrsková, Jitka Tejcová, Ibrahim Bitar, Eva Sládková, Josef Sýkora, Ivan Šubrt

**Affiliations:** 1 Department of Medical Genetics, Faculty of Medicine in Pilsen, Charles University and University Hospital Pilsen, Pilsen, Czech Republic; 2 Biomedical Center, Faculty of Medicine in Pilsen, Charles University, Pilsen, Czech Republic; 3 Department of Microbiology, Faculty of Medicine in Pilsen, Charles University and University Hospital Pilsen, Pilsen, Czech Republic; 4 Department of Pediatrics, Faculty of Medicine in Pilsen, Charles University and University Hospital Pilsen, Pilsen, Czech Republic

**Keywords:** Long-read sequencing, PacBio, Breakpoint analysis, AVPR2 deletion, Nephrogenic diabetes insipidus

## Abstract

**Background:**

X-linked nephrogenic diabetes insipidus (NDI) is a rare genetic renal disease caused by pathogenic variants in the *AVPR2* gene. Single nucleotide variants and small insertions/deletions in *AVPR2* are reliably detected by routine clinical sequencing. Nevertheless, structural variants involving *AVPR2* are challenging to identify accurately by conventional genetic testing. Here, we report a novel deletion of *AVPR2* in a Czech family identified for the first time by targeted long-read sequencing (T-LRS).

**Methods:**

A male proband with X-linked NDI underwent clinical sequencing of the *AVPR2* gene that failed and thus indicated possible whole-gene deletion. Therefore, PCR mapping and subsequent targeted long-read sequencing (T-LRS) using a Pacific Biosciences sequencer were applied to search for the suspected deletion. To validate the deletion breakpoints and prove variant segregation in the family with X-linked NDI, Sanger sequencing of the deletion junction was performed. Quantitative real-time PCR was further carried out to confirm the carrier status of heterozygous females.

**Results:**

By T-LRS, a novel 7.5 kb deletion of *AVPR2* causing X-linked NDI in the proband was precisely identified. Sanger sequencing of the deletion junction confirmed the variant breakpoints and detected the deletion in the probands´ mother, maternal aunt, and maternal cousin with X-linked NDI. The carrier status in heterozygous females was further validated by quantitative real-time PCR.

**Conclusions:**

Identifying the 7.5 kb deletion gave a precise molecular diagnosis for the proband, enabled genetic counselling and genetic testing for the family, and further expanded the spectrum of structural variants causing X-linked NDI. Our results also show that T-LRS has significant potential for accurately identifying putative structural variants.

**Supplementary Information:**

The online version contains supplementary material available at 10.1186/s12920-024-01801-1.

## Background

X-linked nephrogenic diabetes insipidus (X-linked NDI, MIM: 300538) is a rare X-linked recessive disease characterized by the inability of the kidney to concentrate urine in response to the antidiuretic hormone arginine-vasopressin (AVP). The main clinical manifestation is polyuria with polydipsia, failure to thrive, feeding difficulty, and repeated vomiting. Nevertheless, without proper treatment, severe clinical symptoms may develop, such as intellectual disability [1, 2].

X-linked NDI is caused by pathogenic variants in the *AVPR2* gene, which encodes the arginine-vasopressin V2 receptor [3]. AVPR2 is a G-protein coupled receptor that in response to AVP redistributes aquaporin-2 water channel (AQP2) in the renal collecting tubules to make the membrane permeable to water, thereby concentrating the urine [1, 2]. X-linked NDI accounts for approximately 90% of cases with congenital NDI. The remaining 10% of cases are caused by pathogenic variants in the *AQP2* gene with autosomal recessive or dominant inheritance [1, 2] Most pathogenic variants in *AVPR2* are single nucleotide variants [4, 5]. Therefore, the recommended first-tier genetic diagnostic test for individuals with suspected X-linked NDI is sequencing analysis of *AVPR2* [1]. However, non-recurrent deletions of the entire *AVPR2* causing X-linked NDI have also been observed [6]. The reported deletions varied in length but in most cases included not only *AVPR2* but also a neighbouring gene of unknown clinical significance, *ARHGAP4* [6]. Since these deletions, as well as other small structural variants (SVs), are challenging to identify by other clinical testing methods, such as chromosomal microarray or short-read sequencing [7], the next recommended step for patients with suspected large deletions is to perform gene-targeted deletion analysis of *AVPR2* [1].

The most commonly used targeted methods to accurately detect causal large deletions involving *AVPR2* are polymerase chain reaction mapping and Sanger sequencing [6, 8–10]. This approach is labour-intensive [8] and not always applicable. Large deletions and other SVs tend to occur in repetitive genomic regions that are challenging for Sanger and short-read sequencing [11]. However, it has been shown that such difficulties can be overcome by long-read sequencing (LRS) [12–15]. LRS on Pacific Biosciences (PacBio) platforms generates highly accurate long reads (HiFi reads) that can span SVs breakpoints and confidently identify SVs even in difficult-to-sequence regions [11]. Although targeted long-read sequencing (T-LRS) on the PacBio sequencer represents an effective way to accurately detect suspected SV [14], more clinical evidence is needed to confirm the clinical utility of T-LRS.

In this study, we report the first successful application of T-LRS on the PacBio sequencer to identify and fine-map a candidate causal structural variant in a male proband with X-linked NDI. In the proband diagnosed with X-linked NDI based on the clinical manifestations and the family history, genetic cause after clinical sequencing remained unknown, yet suspected. Therefore, we applied further genetic testing to search for suspected whole-gene deletion. Consequently, we precisely detected a novel 7.5 kb deletion of *AVPR2* by T-LRS in the proband, and thus we were able to determine the genetic status of available at-risk relatives in the family and offer genetic testing for other family members that may prevent the birth of affected children or prevent primary manifestation. Our results further demonstrate the utility of T-LRS for accurately discriminating putative causal structural variants.

## Materials and methods

### Clinical description

The proband was born after an uncomplicated pregnancy at 39 + 1 weeks with a birth weight of 3660 g and birth length of 50 cm. Due to significant weight loss, early enteral intake was initiated. At the age of 23 hours, he developed hypernatremic dehydration (the maximum sodium level was 160 mmol/l), which was corrected by intravenous fluid administration (D5%W). The requirement for fluid was high, with a maximum of 300 ml/kg per day. At this time, Hydrochlorothiazide was administered temporarily. The child tolerated oral intake well and clinical symptoms were significantly reduced after targeted therapy. Thus, at 11 days of age, parenteral intake could be ended, and Hydrochlorothiazide was discontinued. Ultrasound showed a congenital duplex left kidney and normal sonography of the heart, liver, gallbladder, pancreas, and spleen. At the age of 19 days, the patient was readmitted to the Neonatology Department for hypernatremic dehydration and Hydrochlorothiazide therapy was restarted. Main symptoms at clinical manifestation were fever, irritability, polyuria, poor feeding and failure to thrive. The analysis of family history revealed the probands maternal uncle was clinically diagnosed with NDI after birth, and two more distant maternal male relatives were monitored and treated by a pediatric nephrologist during childhood and had discontinued treatment in adulthood. Based on the clinical manifestations in the male proband and pedigree analysis a diagnosis of the X-linked NDI was suspected. Other syndromes with polyuria such as Bartter syndrome were contemplated in the process of differential diagnosis, but their type of inheritance was not consistent with the pedigree analysis. The patient was referred for a nephrological follow-up.

Further treatment was based on free water intake, and low salt diet, a thiazide diuretic was given to lower urine output, as needed. The proband stayed in the care of the Department of Pediatrics, University Hospital Pilsen, Czech Republic, until referred to genetic testing by a pediatric nephrologist. Currently, at 8 years of age, the proband is treated with Hydrochlorothiazide, Verospiron, and KCl. His daily fluid intake is 4000–5000 ml.

### DNA isolation

Genomic DNA was isolated from peripheral blood of the proband and his available family members using the Gentra Puregene Blood Kit (Qiagen, Hilden, Germany) according to the manufacturer’s protocol. The concentration and purity of the DNA were assessed using a spectrophotometer DeNovix DS-11 FX (DeNovix, Wilmington, DE).

### PCR and Sanger sequencing

The entire coding sequence with the flanking intronic regions of the *AVPR2* gene (clinical sequencing) and the breakpoint junction (breakpoint sequencing) were amplified and sequenced in the proband using primers listed in Table S1 (Additional file 1). Breakpoint sequencing was also performed on available family members for segregation analysis. Genomic regions were amplified using AmpliTaq Gold 360 Master Mix (Thermo Fisher Scientific, Waltham, MA) in a final 10 μl PCR mixture containing 1 μl of H_2_O, 5 μl of AmpliTaq Gold 360 Master Mix, 2 μl of 10 μM forward and reverse primer mix and 2 μl of DNA (35 g/μl) according to the manufacturer’s protocols. PCR products were purified using Agencourt AMPure XP magnetic beads (Beckman Coulter, Brea, CA) and sequencing reactions were performed using the Gerbera Sequencing Kit v3.1 (SEQme, Dobříš, Czech Republic) according to the manufacturer’s protocols. Sequencing products were purified using CleanSEQ (Beckman Coulter, Brea, CA) and separated on the ABI PRISM 3130 Genetic Analyser (Thermo Fisher Scientific, Waltham, MA). Sequencing data were analysed using BioEdit sequence alignment editor v.7.0.5.3 [16].

### PCR mapping

Several genomic regions flanking the deletion in the proband were amplified using AmpliTaq Gold 360 Master Mix (Thermo Fisher Scientific, Waltham, MA) in a final 10 μl PCR mixture containing 1 μl of H_2_O, 5 μl of AmpliTaq Gold 360 Master Mix, 2 μl of 10 μM forward and reverse primer mix and 2 μl of DNA (35 ng/μl) according to the manufacturer’s protocols. Primer sequences can be found in Table S1 (Additional file 1). PCR products were analysed by agarose gel electrophoresis (2%).

### Targeted long-read sequencing and analysis

The selected target region in the proband was amplified by long-range PCR using the Phusion High-Fidelity PCR Kit (Thermo Fisher Scientific, Waltham, MA) in a final 50 μl PCR mixture containing 34 μl H_2_O, 10 μl of 5× Phusoin GC Buffer, 1 μl of 10 μM dNTPs, 2.5 μl of 10 μM forward and reverse primer mix, 2 μl of DNA (50 ng/μl) and 0.5 μl Phusion DNA Polymerase. Primer sequences can be found in Table S1 (Additional file 1). The PCR protocol was as follows: Initial denaturation at 98 °C for 30 s, 30 cycles of 98 °C for 5 s, 63.5 °C for 15 s and 72 °C for 35 s and final extension at 72 °C for 10 min. The amplified products (~ 3900 bp long) were verified by agarose gel electrophoresis (1%) and purified using 0.5× Agencourt AMPure XP magnetic beads (Beckman Coulter, Brea, CA). These amplicons were subjected to SMRTbell library preparation and sequenced on a Pacific Biosciences Sequel I system (Pacific Biosciences, Menlo Park, CA) as recommended by Pacific Biosciences. To generate highly accurate long reads (Hifi reads), Circular Consensus Sequence analysis was performed using SMRT Link v.10.1. HiFi reads were aligned to the reference human genome (hg19) using minimap2 v.2.1 [17] with default parameters and visualized in Integrative Genomics Viewer v.12.2.3 [18]. The flanking sequences of the breakpoints were manually evaluated for the presence of microhomology or repetitive elements.

### Quantitative real-time PCR

The copy number of the AVPR2 gene in available healthy females was determined by quantitative real-time PCR on Rotor-Gene Q (Qiagen, Germantown, MD) in a final 25 μl PCR mixture containing 12.5 μl of ABsolute QPCR Mix, no ROX (Thermo Fisher Scientific, Waltham, MA), 1 μl of 10 μM forward primer and 1 μl of 10 μM reverse primer, 1.2 μl 20× EvaGreen Dye (Biotium, Freemont, CA), 2.5 μl of DNA (10 ng/μl) and 6.8 μl H_2_O. Primer sequences can be found in Table S1 (Additional file 1). Each sample was tested in three technical replicates with the following conditions: Initial denaturation at 95 °C for 15 min, 40 cycles of 95 °C for 15 s and 60 °C for 60 s and melting curve analysis at 60–90 °C/step 0.5 °C. qPCR data were analysed with double delta Ct analysis, and the copy number of *AVPR2* was normalized to the copy number of the *GAPDH* reference gene in the same sample. The relative copy number of *AVPR2* to an unrelated control female were compared and shown in the graph for each sample.

## Results

### Clinical genetic analysis

**Fig. 1:**
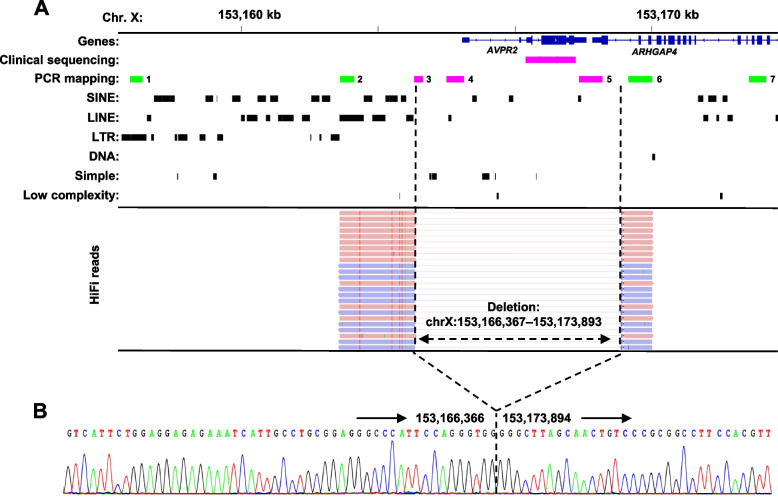
Identification of *AVPR2* deletion. **A** Schematic presentation showing (from top to bottom) the position of genes, amplicons for clinical sequencing of *AVPR2,* amplicons for PCR mapping, and repetitive sequences in the RepeatMasker track. Amplified amplicons are coloured green, not amplified magenta. PacBio HiFi reads of long-range amplicons aligned to the human genome reference sequence (hg19) showing the unambiguous 7526 bp deletion (chrX:153,166,367–153,173,893) are shown at the bottom. Separate alignments from the same read are connected by a thin line. **B** Sanger sequencing of the breakpoint junction confirming the deletion breakpoints. The breakpoint junction is indicated by a dashed line

The proband with the clinical diagnosis of X-linked NDI underwent clinical sequence analysis of the *AVPR2* gene. However, PCRs covering coding exons and flanking intronic regions of *AVPR2* failed to amplify (Fig. 1A), which suggested a large deletion covering the entire *AVPR2*.

### Identification of deletion

To refine and confirm the suspected deletion, we designed several primer pairs to produce amplicons surrounding *AVPR2* within the region with previously published deletions [6]. Only PCR amplicons 1, 2, 6, and 7 were successfully produced (Fig. 1A), which indicates a deletion covering the entire *AVPR2* and the last exon of adjacent *ARHGAP4*. To identify the size and location of the deletion at single nucleotide resolution in the region containing multiple repetitive elements, we applied T-LRS. Targeted amplicons overlapping the deletion were obtained by long-range PCR and subsequently sequenced on the PacBio sequencer. As a result, generated HiFi reads aligned to the human reference hg19 revealed a 7526 bp deletion of chrX:153,166,367–153,173,893 (Fig. 1A). The 5′ breakpoint was located near a repetitive element L1ME1 in the intergenic region between *AVPR2* and adjacent *L1CAM*, while the 3′ breakpoint was delineated in intron 22 of *ARHGAP4* with no repetitive elements. The breakpoint regions show no microhomologies.

### Confirmation of deletion and segregation analysis

**Fig. 2:**
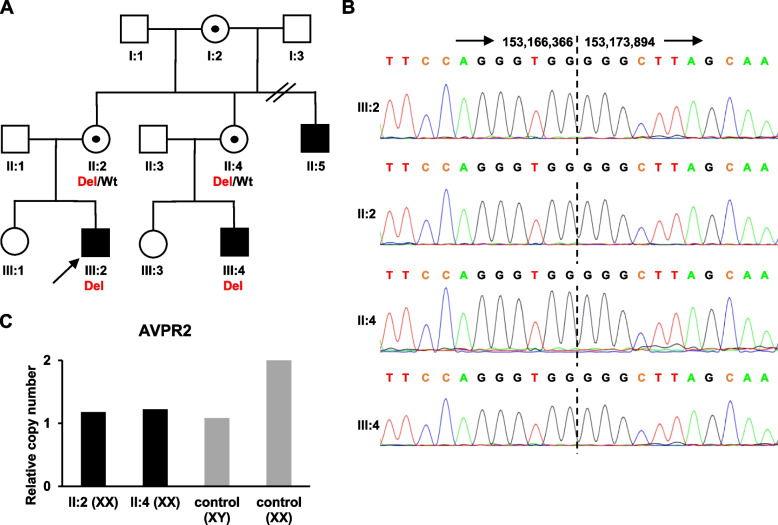
Family pedigree and segregation analysis. **A** Pedigree of the family with X-linked NDI and segregation of the 7.5 kb deletion (chrX:153,166,367–153,173,893). Available genotypes are shown below symbols: Del – deletion allele, Wt – wild-type allele. **B** Sanger sequencing of the breakpoint junction in the proband and available family members. The breakpoint junction is indicated by a dashed line. **c** qPCR data showing the relative copy number of *AVPR2* in suspected female carriers (II:2 and II:4) and the control male to the copy number in the control female

The precise deletion breakpoints in the proband identified by T-LRS were validated by Sanger sequencing with PCR primers amplifying the breakpoint junction (Fig. 1B). Additionally, Sanger sequencing of the breakpoint junction in available at-risk family members (Fig. 2A) detected the identical deletion in a younger maternal cousin (III:4) also affected with X-linked NDI and in healthy females (II:2 and II:4) (Fig. 2B). To confirm the carrier status of the healthy females (II:2 and II:4), we performed copy number analysis of *AVPR2* using quantitative real-time PCR (qPCR). The relative copy number of *AVPR2* in all tested females was one copy that correlated with the hemizygous control male (Fig. 2C) and thus proved the heterozygous status of these females.

## Discussion

Timely and accurate diagnosis of X-linked NDI is beneficial for patients because untreated patients can develop severe symptoms. The diagnostic steps of X-linked NDI reflect the main clinical symptoms of the disease. However, early diagnosis of X-linked NDI can be clinically challenging as symptoms may not be specific [8]. Therefore, molecular genetic analysis of the *AVPR2* gene is important to confirm the diagnosis at the molecular level. Identification of genetic cause of X-linked NDI in the proband also enables genetic testing for other family members to achieve the birth of unaffected child or to prevent primary manifestation. However, in some cases, widespread clinical sequencing of *AVPR2* reveals difficult-to-confirm structural variants. Hence, there is a need to use better tools to provide a precise diagnosis. Here, we identified a novel pathogenic 7.5 kb deletion of *AVPR2* in a family with X-linked NDI using T-LRS on the PacBio sequencer.

In our case, clinical sequence analysis of *AVPR2* in the proband with clinically suspected X-linked NDI indicated a large deletion of the entire *AVPR2*. In contrast to previous studies using even thirty-two primer pairs around *AVPR2* to characterize the large deletions [8], we performed only several PCRs. The approximately defined region was subsequently amplified and sequenced on the PacBio sequencer. As in previous studies using LRS [13, 15], T-LRS generated highly accurate sequence data even in the difficult-to-sequence regions (repetitive elements and homopolymer sequences) and precisely identified the deletion breakpoints. Detection of the deletion breakpoints was crucial for a complete diagnosis of the proband and for identifying related asymptomatic heterozygous female carriers who might otherwise stay undiagnosed, as routine clinical sequencing of *AVPR2* gene would be falsely negative. Using precisely identified deletion breakpoints in the proband, we were able to design PCR primers to amplify the breakpoint junction. Sanger sequencing of the breakpoint junction in the proband demonstrated the accuracy of HiFi reads and segregation of the deletion in other family members based on X-linked recessive inheritance.

Since the deletion breakpoints were not mapped to be within repetitive elements and no sequence homology was found at the junction, proposed mechanisms for such deletion are non-homologous end joining or fork stalling and template switching [6, 19]. Both breakpoints lay in the previously defined regions with more breakpoints in which deletion is probably stimulated by the local genome architecture [6]. We report the first deletion within these two regions, which can contribute to elucidating the mechanisms of non-recurrent deletions causing X-linked NDI.

In addition to the entire *AVPR2*, the 7.5 kb deletion encompassed the last exon (exon 22) of the adjacent *ARHGAP4* gene. This is consistent with previous results where all but one [20] of the previously published large deletions causing X-linked NDIs included a part or all of the *ARHGAP4* gene [6, 8–10, 21–30]. Despite lacking the part of *ARHGAP4*, both patients in this family had no symptoms other than those associated with NDI [1] which supports the results of the majority of previous studies [6, 8, 21–24, 26–29]showing that, in such cases, disruptions of *ARHGAP4* do not lead to a different clinical phenotype.

## Conclusions

In summary, the identification of the novel 7.5 kb deletion enabled a precise molecular diagnosis for the proband, genetic counselling and genetic testing for at-risk family members, and further expanded the spectrum of SVs causing X-linked NDI. Our results also support a new strategic workflow for identifying pathogenic variants in rare disease cases [7]. According to this strategy, when a single-gene disease is suspected, targeted approaches are recommended. If Sanger sequencing data indicate the possibility of SV, LRS is then advised. We hope that the further application of T-LRS will help to identify and confirm the suspected causative SVs also in other genetic diseases.

### Supplementary Information

**Additional file 1.**

## Data Availability

The datasets used and/or analysed during the current study are available from the corresponding author on reasonable request.
