# Supplementary material for: Targeted long-read sequencing identified a causal structural variant in X-linked nephrogenic diabetes insipidus
Source: BMC Med Genomics. 2024 Jan 22;17:29. doi: 10.1186/s12920-024-01801-1 (PMC10804598; doi:10.1186/s12920-024-01801-1)
Supplement: Supplementary file 1 — Additional file 1. [file 12920_2024_1801_MOESM1_ESM.docx]

**Table S1.** List of primers used in this study.

| **Experiment** | **Oligo name** | **Sequence** |
| --- | --- | --- |
| Clinical sequencing | AVPR2_ex2_F | ATCCGTCTGTCTGACCATCC |
|  | AVPR2_ex2_R | GGTTAGAGAGGGTGCTGTGC |
|  | AVPR2_ex3_F | CTGCTAGGAGCCAGGAAGTG |
|  | AVPR2_ex3_R | AGGGCAATCCAGGTGACATA |
|  | AVPR2_ex4_F | CGGGGTCACTGACTGCTG |
|  | AVPR2_ex4_R | AGGCAGCTGAGCTTCTCAAA |
| PCR mapping | Amplicon1_F | CCACATCACCCAGGAAGTCT |
|  | Amplicon1_R | ATTGGGTGTGCTCACATGAA |
|  | Amplicon2_F | AGGCAGAGACTGTTGGGCTA |
|  | Amplicon2_R | GTCTGTGTTGGTTGGGGTTC |
|  | Amplicon3_F | AGAGAAATCATTGCCTGCGGA |
|  | Amplicon3_R | TGAGGGCCGTACCCATAGAT |
|  | Amplicon4_F | TGGGTCGCACCATTTCTCTC |
|  | Amplicon4_R | CTCTGGGATGCAAGAAGCCA |
|  | Amplicon5_F | CCCAGCTGTATGAGGAGAGC |
|  | Amplicon5_R | CCTAGACACGACCCCCAAG |
|  | Amplicon6_F | ACAAATGCCAGCCTTGAATC |
|  | Amplicon6_R | GAAGGAGCACTCGAAACAGG |
|  | Amplicon7_F | CTGTACAGCCTGCTGTTGGA |
|  | Amplicon7_R | GCATCCCAGAGAGAAAGGTG |
| Long-range PCR | Amplicon2_F | AGGCAGAGACTGTTGGGCTA |
|  | Amplicon6_R | GAAGGAGCACTCGAAACAGG |
| Breakpoint sequencing | AVPR2_BP_F | TAGGTAACAGGCGGTGCC |
|  | AVPR2_BP_R | GATCAGTGGTTGCTAGGCTTTC |
| Quantitative Real-Time PCR | AVPR2_qrt_F | GGGCCTTCTCGCTCCTTCT |
|  | AVPR2_qrt_R | AGGGCAATCCAGGTGACATAG |
|  | GAPDH_qrt_F | TCCTCTGACTTCAACAGCGAC |
|  | GAPDH_qrt_R | CGCCAGACCCTGCACTTTTTA |
